# Children and Crime

**Published:** 1888-09-08

**Authors:** 


### The Hospital

A Weekly Institutional Journal of

Science, Medicine, Nursing, and Philanthropy.

*For Hospitals, Asylums, Medical Practitioners, Students, Nurses, Families, Parishes, Congregations, and the Charitable Public.*

Vol. IV. No. 102.] [September 8, 1888.

### Children and Crime.

Childhood, almost more than any other period of life, owes a deep debt of gratitude to modern scientific medicine. It is not only that powders are made palatable which were formerly nauseous, and that mixtures are so pleasant that children will ask for them. These are mere trifles. But, thanks to the doctor, the nurse is now a reasonable woman, the dressmaker must reserve her fashionable and ridiculous garments for grown-up clients, the schoolmaster uses his brains and his tongue more than his cane, and even lawyers and magistrates are beginning to see that reason must be mixed with law, and that science is of more authority than the most pompous exposition of statutes that are sometimes antiquated and not seldom absurd.

"Children and Crime"—the mere juxtaposition of the two words makes a thoughtful man sad. Why should children ever be criminals? Is the explanation to be sought in regions theological; and are we to trace juvenile criminality back to original sin and to Adam? Assuredly not! Crime is not natural to children; it is most unnatural. It is no more natural to infant human beings than to infant sheep, infant horses, or infant elephants. There is in all young, as in all adult life—a series of wants and a set of tendencies to action. The wants and the tendencies to action are both in themselves perfectly natural and perfectly harmless. The environments in which normal infancy ought to find itself, and in which Nature intended that it should find itself, are such as to provide for its wants and to give room for the free exercise of its tendencies. Granted such normal and suitable environments, child crime would become practically a thing unknown.

The British Medical Association has appointed a commission "to conduct an investigation as to the average development and condition of brain function among children in primary schools." In connexion with this, the *British Medical Journal* gives a practical illustration of the necessity for some such investigation from the Hammersmith Police Court. Seven boys, whose ages varied from eleven to thirteen years, were charged with being concerned with breaking into certain premises and stealing money. Two of them were committed to prison for one month, with hard labour, and one was ordered to have six strokes of the birch. The story of child crime which we print in another place is still more terrible. These are mere samples of cases which are occurring constantly. Large towns breed these young thieves and murderers, and the larger the town the more successful is it as a breeding-place. London probably has a worse eminence in this respect than any other ci y in the world.

But, happily, we are not comfortable under these dreadful and disgraceful circumstances. A considerable proportion of the British public are ashamed of a good many things for which as citizens they are more or less responsible; and the condition of the child-population in certain districts is one of the things that make the face burn with shame and anger. We have already indicated that practically the children themselves are not responsible. They are what generations of hereditary influence and a feebly intelligent and ill-administered social system has made them. In some departments of the State we are too much governed by half, in others we are not governed at all. Carlyle longed for a strong despotism, provided it was both intelligent and animated by noble motives. That longing may well find an echo in the heart of every one who sees human fields which might have borne abundant crops of intellectual and moral grain handed over to the devil of desolation and moral ruin.

The saddle must be put on the right horse or horses. Child criminals are what they are primarily because of their parentage. They are born to an inheritance of brutality, ignorance, and cunning. Exactly as the wild beast that has attained a certain age has to enter upon the struggle for survival, and by keenness of wit, swiftness of foot, or sharpness of tooth, must hold his own with other wild beasts similarly endowed to himself, so must the child of certain parents obey his natural instincts under the only conditions which are possible for him—conditions of theft and violence, of escape or capture, of punishment at home if he fails, of punishment in prison or elsewhere if he succeeds.

But the worthless and degraded parents are not alone to blame. The social system must accept its share of responsibility, and it is very much the larger share. What is the "social system"? Do not let us delude ourselves by thinking of it as an entity outside ourselves, an entity upon which we may put all our failures and social immoralities. The social system consists of the individuals who together constitute the population of the country or town, and of their laws and manners. We are our social system; our fathers before us were their social system. That which happens among us is the harvest of our own or our fathers' sowing. Child-crime, in all its hardness, in all its viciousness, in all its hopelessness, is to a large extent the work of our own hands.

If we have made, can we unmake? If the social system has been powerful enough to fill our reformatories and to partly people our gaols with child criminals, can the social system do anything to empty the gaols and to prevent the up-springing and growth of those who should never see the inside of a prison at all? If people would but take the trouble to understand the problem and assure themselves that sixpence taken out of the left-hand pocket is exactly equivalent to the same sum taken out of the right-hand pocket, the thing might be done. The money spent on prisons should be spent on schools. That is where the whole secret lies. But, say some, "the money has been spent on schools, and what is the result?" The money has not been spent wisely enough as yet. Schools and teachers have been feeling their way, and have not yet got into the right track. The teachers are not of the right class. They have been men of arithmetic, of writing, of spelling, and of routine. They should have been men of physiology, of medical experience, of sense, and of humanity as well. We have sent inspectors to the slums to see if the poor little human wrecks could be steered through the shoals and quicksands of the sixth standard; but we have sent no inspectors down to inquire whether any of them had water in the head, ricketty joints, muscles so poor and flabby that they could hardly run, and brains so ill-nourished that memory was impossible.

We are surely men of sense. We do not expect an ill-fed cab-horse to draw a brewer's dray, or a fat alderman to run ten miles an hour. In ordinary circumstances we use fairly reasonable means in order to secure our ends. But in dealing with the children of criminal parents, we have displayed exactly the same kind of wisdom as a man would who should expect that because ships are made of oak, he could secure a whole fleet by sowing plenty of acorns. Let any man ask himself what connexion there is between arithmetic and truthfulness, between good spelling and honesty? Is there any necessary connexion at all? But the connexion between a cold skin and a warm jacket, between hunger and a hot potato from the potato merchant's stall, is very obvious to a small boy, or even to a red-tape educator.

What, then, is a practical method of dealing with child criminals? The sending of them to prison is the maximum of stupidity and inefficiency. They should be at once and completely separated from their present surroundings and influences. They should be well fed, sufficiently clothed, and warmly housed. They should be given the elements of education, a reasonable moral training, and be taught a trade or calling by which they could live. Their period of apprenticeship and restraint should be of sufficient duration to clear out from habit, and, if possible, from memory, all old associations of every kind. All this should be done by and under the supervision of men of sense, of reasonable kindness, of adequate medical and scientific knowledge. In fine, the same degree of reason and good sense, should be used here as is commonly displayed by individuals in the conduct of their own business. That is to say we must cultivate our land efficiently, and sow good and right seed if we are to expect a full and profitable crop.

